# CNV-Finder: streamlining copy number variation discovery

**DOI:** 10.1093/bioadv/vbag205

**Published:** 2026-07-22

**Authors:** Nicole Kuznetsov, Kensuke Daida, Mary B Makarious, Bashayer Al-Mubarak, Kajsa Atterling Brolin, Laksh Malik, Cedric Kouam, Breeana Baker, Raquel Real, Kathryn Step, Lara M Lange, Lesley Wu, Miriam Ostrozovicova, Katherine M Andersh, Pin-Jui Kung, Yasser Mecheri, Yi-Wen Tay, Behloul Soundous Malek, Nada Al Tassan, Maria Teresa Periñan, Samantha Hong, Mathew J Koretsky, Lana Sargeant, Kristin Levine, Cornelis Blauwendraat, Kimberley J Billingsley, Sara Bandres-Ciga, Hampton L Leonard, Soraya Bardien, Huw R Morris, Andrew B Singleton, Mike A Nalls, Dan Vitale

**Affiliations:** Center for Alzheimer’s and Related Dementias (CARD), National Institute on Aging and National Institute of Neurological Disorders and Stroke, National Institutes of Health, Bethesda, MD 20892, United States; DataTecnica LLC, Washington, DC 20037, United States; Center for Alzheimer’s and Related Dementias (CARD), National Institute on Aging and National Institute of Neurological Disorders and Stroke, National Institutes of Health, Bethesda, MD 20892, United States; Center for Alzheimer’s and Related Dementias (CARD), National Institute on Aging and National Institute of Neurological Disorders and Stroke, National Institutes of Health, Bethesda, MD 20892, United States; DataTecnica LLC, Washington, DC 20037, United States; Department of Clinical and Movement Neurosciences, Queen Square Institute of Neurology, University College London, London WC1N 3BG, United Kingdom; Center for Genomic Medicine, King Faisal Specialist Hospital and Research Center, Riyadh 12713, Saudi Arabia; Translational Neurogenetics Unit, Department of Experimental Medical Science, Lund University, Lund 221 00, Sweden; Centre for Preventive Neurology, Wolfson Institute of Population Health, Queen Mary University of London, London E1 4NS, United Kingdom; Center for Alzheimer’s and Related Dementias (CARD), National Institute on Aging and National Institute of Neurological Disorders and Stroke, National Institutes of Health, Bethesda, MD 20892, United States; Center for Alzheimer’s and Related Dementias (CARD), National Institute on Aging and National Institute of Neurological Disorders and Stroke, National Institutes of Health, Bethesda, MD 20892, United States; Center for Alzheimer’s and Related Dementias (CARD), National Institute on Aging and National Institute of Neurological Disorders and Stroke, National Institutes of Health, Bethesda, MD 20892, United States; Department of Clinical and Movement Neurosciences, Queen Square Institute of Neurology, University College London, London WC1N 3BG, United Kingdom; Division of Molecular Biology and Human Genetics, Faculty of Medicine and Health Sciences, Stellenbosch University, Cape Town 7505, South Africa; South African Medical Research Council Centre for Tuberculosis Research, Stellenbosch University, Cape Town 7505, South Africa; Laboratory of Neurogenetics, National Institute on Aging, National Institutes of Health, Bethesda, MD 20892, United States; Institute of Neurogenetics, University of Luebeck, Luebeck 23562, Germany; Department of Clinical and Movement Neurosciences, Queen Square Institute of Neurology, University College London, London WC1N 3BG, United Kingdom; Department of Neurology, P.J. Safarik University, Kosice 04011, Slovak Republic; Department of Neurology, University Hospital of L. Pasteur, Kosice 04011, Slovak Republic; Department of Neuromuscular Diseases, UCL Queen Square Institute of Neurology, London WC1N 3BG, United Kingdom; Center for Alzheimer’s and Related Dementias (CARD), National Institute on Aging and National Institute of Neurological Disorders and Stroke, National Institutes of Health, Bethesda, MD 20892, United States; Genome and Systems Biology Degree Program, National Taiwan University and Academia Sinica, Taipei 106319, Taiwan; Neurology Department, Benbadis University Hospital, Constantine 25018, Algeria; Department of Biomedical Science, Faculty of Medicine, University of Malaya, Kuala Lumpur 50603, Malaysia; Neurology Department, Benbadis University Hospital, Constantine 25018, Algeria; Center for Genomic Medicine, King Faisal Specialist Hospital and Research Center, Riyadh 12713, Saudi Arabia; Centre for Preventive Neurology, Wolfson Institute of Population Health, Queen Mary University of London, London E1 4NS, United Kingdom; Unidad de Trastornos del Movimiento, Servicio de Neurología y Neurofisiología Clínica, Hospital Universitario Virgen del Rocío, Instituto de Biomedicina de Sevilla, CSIC, Universidad de Sevilla, Seville 41013, Spain; Center for Alzheimer’s and Related Dementias (CARD), National Institute on Aging and National Institute of Neurological Disorders and Stroke, National Institutes of Health, Bethesda, MD 20892, United States; Center for Alzheimer’s and Related Dementias (CARD), National Institute on Aging and National Institute of Neurological Disorders and Stroke, National Institutes of Health, Bethesda, MD 20892, United States; DataTecnica LLC, Washington, DC 20037, United States; Center for Alzheimer’s and Related Dementias (CARD), National Institute on Aging and National Institute of Neurological Disorders and Stroke, National Institutes of Health, Bethesda, MD 20892, United States; Laboratory of Neurogenetics, National Institute on Aging, National Institutes of Health, Bethesda, MD 20892, United States; School of Nursing, Virginia Commonwealth University, Richmond, VA 23298, United States; Center for Alzheimer’s and Related Dementias (CARD), National Institute on Aging and National Institute of Neurological Disorders and Stroke, National Institutes of Health, Bethesda, MD 20892, United States; DataTecnica LLC, Washington, DC 20037, United States; Center for Alzheimer’s and Related Dementias (CARD), National Institute on Aging and National Institute of Neurological Disorders and Stroke, National Institutes of Health, Bethesda, MD 20892, United States; Laboratory of Neurogenetics, National Institute on Aging, National Institutes of Health, Bethesda, MD 20892, United States; Center for Alzheimer’s and Related Dementias (CARD), National Institute on Aging and National Institute of Neurological Disorders and Stroke, National Institutes of Health, Bethesda, MD 20892, United States; Center for Alzheimer’s and Related Dementias (CARD), National Institute on Aging and National Institute of Neurological Disorders and Stroke, National Institutes of Health, Bethesda, MD 20892, United States; Center for Alzheimer’s and Related Dementias (CARD), National Institute on Aging and National Institute of Neurological Disorders and Stroke, National Institutes of Health, Bethesda, MD 20892, United States; DataTecnica LLC, Washington, DC 20037, United States; German Center for Neurodegenerative Diseases (DZNE), Tübingen 72076, Germany; Centre for Genetic Epidemiology, Institute for Clinical Epidemiology and Applied Biometry, University of Tübingen, Tübingen 72074, Germany; Division of Molecular Biology and Human Genetics, Faculty of Medicine and Health Sciences, Stellenbosch University, Cape Town 7505, South Africa; South African Medical Research Council Centre for Tuberculosis Research, Stellenbosch University, Cape Town 7505, South Africa; Stellenbosch University Genomics of Brain Disorders Research Unit, South African Medical Research Council, Stellenbosch University, Cape Town 7599, South Africa; Department of Clinical and Movement Neurosciences, Queen Square Institute of Neurology, University College London, London WC1N 3BG, United Kingdom; Center for Alzheimer’s and Related Dementias (CARD), National Institute on Aging and National Institute of Neurological Disorders and Stroke, National Institutes of Health, Bethesda, MD 20892, United States; Laboratory of Neurogenetics, National Institute on Aging, National Institutes of Health, Bethesda, MD 20892, United States; Center for Alzheimer’s and Related Dementias (CARD), National Institute on Aging and National Institute of Neurological Disorders and Stroke, National Institutes of Health, Bethesda, MD 20892, United States; DataTecnica LLC, Washington, DC 20037, United States; Center for Alzheimer’s and Related Dementias (CARD), National Institute on Aging and National Institute of Neurological Disorders and Stroke, National Institutes of Health, Bethesda, MD 20892, United States; DataTecnica LLC, Washington, DC 20037, United States

## Abstract

**Motivation:**

Copy Number Variations (CNVs) play pivotal roles in complex disease etiology, often requiring large sample sizes to analyze disease associations. While genotyping arrays offer a cost-effective approach for CNV detection using Log R Ratio (LRR) and B Allele Frequency (BAF) signals, existing independent array-based callers suffer from high false positive rates and noise susceptibility, burdening manual validation.

**Results:**

We present CNV-Finder, a deep learning pipeline employing Long Short-Term Memory (LSTM) networks for large-scale CNV identification within user-defined genomic regions. Trained on expert-annotated samples from the Global Parkinson’s Genetics Program across four neurodegenerative disease-associated genes (*PRKN, LINGO2, MAPT, SNCA*), CNV-Finder integrates human feedback to iteratively improve performance. In benchmarking across 105 936 samples spanning 11 ancestries and nearly 150 cohorts, the model achieved 91% and 89% visual confirmation rates for *PRKN* deletions and duplications at high-confidence thresholds. In two validation cohorts, CNV-Finder nominated 83% fewer candidates than a popular Hidden Markov Model-based caller while maintaining higher confirmation rates. Validation through MLPA, short-read, and long-read sequencing demonstrated robust performance, generalizing to diverse signatures including homozygous deletions and *SNCA* triplications absent from training. Our findings highlight human expertise’s value in complex loci like 17q21.31.

**Availability and implementation:**

CNV-Finder is freely available at https://github.com/nvk23/CNV-Finder.

## 1 Introduction

### 1.1 Copy number variation detection in disease genetics

Copy Number Variations (CNVs) are structural variants (SVs) that play key roles in the diversity of genetic architecture and disease risk. Spanning hundreds to hundreds of thousands of base pairs, these genomic variations, including duplications, deletions, insertions, and complex rearrangements, deviate from the expected number of copies within DNA segments (Pös *et al*. 2021). Given the growing interest in understanding the contribution of SVs to disease heritability and susceptibility, these variants have been examined across multiple genetic modalities, including genotyping arrays, short-read, and long-read whole genome sequencing (WGS). Among these approaches, genotyping arrays are a cost-effective and widely accessible method for diverse, large-scale investigations of genetic data. While whole genome sequencing is increasingly feasible at scale, genotyping arrays remain particularly well-suited for global, multi-ancestry initiatives and have enabled the identification of clinically relevant CNVs across large, diverse cohorts (Aguirre *et al*. 2019, Collins *et al*. 2022, Auwerx *et al*. 2024). This is especially relevant when investigating rare CNVs, where maximizing sample size across diverse phenotypes is critical to achieving sufficient power in downstream carrier analyses. CNV detection in arrays is facilitated by evaluating normalized signal intensity metrics, Log R Ratio (LRR) and B Allele Frequency (BAF) (Lin *et al*. 2013, Lavrichenko *et al*. 2021, Pös *et al*. 2021). The ability to detect CNVs and accurately determine their size depends on the genotyping array’s probe density across the region of interest. Enhancing the sensitivity and mitigating false positive rates of an array-based SV calling algorithm can expedite the identification of accurate candidates by significantly minimizing the number of samples that require laborious manual review.

Popular computational approaches for array-based CNV detection have largely relied on Hidden Markov Models (HMMs), which model changes between copy number states across genomic positions to infer CNV boundaries (Colella *et al*. 2007, Wang *et al*. 2007). While HMMs have been widely adopted, they are susceptible to noise in signal intensity data or areas of low probe density, making them prone to exhibiting high false positive rates in these regions (Diskin *et al*. 2008, Lin *et al*. 2011). Ensemble approaches that integrate multiple SV callers, such as these HMM-based algorithms, have been shown to improve recall; however, these frameworks are inherently limited by the performance of their weakest constituent model, showing an increase in reported false positives due to the lack of consensus between multiple callers and introducing additional computational overhead from reliance on results from other pipelines (van Baardwijk *et al*. 2024). Therefore, there is still a continued need for accurate, independent calling algorithms that can perform reliably and efficiently on their own.

Although some array-based callers report CNV positional boundaries, they are often constrained by probe spacing rather than true breakpoints, typically requiring validation through sequencing-based methods for accurate boundary characterization (Dellinger *et al*. 2010). For the purpose of investigating disease relationships, this level of resolution is rarely the primary objective. Instead, individual-level CNV carrier status in a gene or region of interest enables genotype-phenotype analyses that inform our understanding of the contribution of structural variation to disease (Bailey *et al*. 2002, Stankiewicz and Lupski 2002, Carvill and Mefford 2013). Achieving the specificity required for such analyses at scale remains a persistent challenge, particularly when balancing sensitivity against the burden of manual validation. Here we present CNV-Finder, a machine learning-based tool designed for targeted CNV screening using genotyping array data. By focusing on user-defined regions of interest and prioritizing carrier identification over exact breakpoint resolution, CNV-Finder efficiently pinpoints high-confidence candidates for downstream disease association studies or further investigation via higher-resolution methods such as long-read sequencing. In doing so, it substantially reduces the manual validation and cost burden associated with large-scale CNV discovery.

### 1.2 Genes of interest

To showcase one practical application, we utilized the biobank-scale resource of the Global Parkinson’s Genetics Program (GP2), building and applying CNV-Finder models to genes previously implicated in neurodegenerative disease (NDD) risk as a means of validating the tool’s performance at scale. Model development included deletions and duplications in *PRKN* (chr6:161 347 557–162 727 802 [hg38]), deletions in *LINGO2* (chr9:27 948 085–29 213 000 [hg38]), and duplications in *MAPT* (chr17:45 894 381–46 028 333 [hg38]). These intervals exhibited sufficiently high rates of deletions and duplications to allow for the creation of large, robust training sets for both CNV types. The variants in these regions were well-defined, minimizing noise and ensuring consistency among manual classifications. *SNCA* (chr4:89 724 098–89 838 296 [hg38]) was included to demonstrate the final models’ usability on previously unseen genes with markedly lower frequencies.

Bi-allelic *PRKN* structural variants are an established cause of autosomal recessive Parkinson’s disease (PD), though the significance of single heterozygous duplications and deletions in PD risk remains uncertain due to incomplete CNV data and conflicting findings in the literature (Huttenlocher *et al*. 2015, Zhu *et al*. 2022, Ahmad *et al*. 2023). *LINGO2* SVs have been similarly implicated in essential tremor and PD, with conclusions limited by sample size (Vilariño-Güell *et al*. 2010, Soraya *et al*. 2021). *MAPT* is a complex locus characterized by high linkage disequilibrium, distinct functional haplotypes, and a common inversion among Europeans (de Jong *et al*. 2012). The H1 haplotype of *MAPT* is associated with PD and other tauopathies like Corticobasal Degeneration (CBD), which may present clinically as Corticobasal Syndrome (CBS) (Zhang *et al*. 2017). The broader 17q21.31 region, including neighboring genes such as *KANSL1*, is implicated in Progressive Supranuclear Palsy (PSP), Frontotemporal Dementia (FTD), Dementia with Lewy Bodies (DLB), and Alzheimer’s disease (AD) (Jun *et al*. 2016, Wang *et al*. 2022). Duplications and triplications in *SNCA* have been strongly linked to monogenic PD (Pankratz and Foroud 2007, Labbé *et al*. 2016). The resulting pre-trained models are a publicly available resource to enable large-scale, parallelized CNV screening across multiple cohorts, phenotypes, and ancestries beyond these selected regions.

## 2 Methods

### 2.1 Genotyping data inputs

All samples featured in both model training and testing underwent genotyping using the Illumina NeuroBooster array (NBA) (Bandres-Ciga *et al*. 2024) and subsequent processing by the Global Parkinson’s Genetics Program (GP2) (Global Parkinson’s Genetics Program 2021), although CNV-Finder is agnostic to the array platform and to any particular processing pipeline. The NBA was chosen for its specific optimization for NDDs, therefore improving coverage in the regions we are analyzing and enhancing reliability in signal intensity measures. Notably, around 1.9 million variants are included in the array, with over 95 000 variants specific to the context of neurological disorders. Regardless of array used, the position, chromosome, BAF, and LRR values for all single nucleotide-polymorphism (SNPs) serves as the necessary parameters for the pipeline. We incorporated all available probe intensity data, including instances where a single SNP was captured by multiple probes, as well as any CNV-specific probes on the array. Because these duplicate and CNV-targeted probes only make up a small fraction of the input file and our algorithm relies on aggregated measures across many variants, retaining only one probe per chromosome position has minimal impact on results. To enhance data quality, users have the option to specify a customizable GenTrain score cut-off, which defaults to 0.2, thus filtering out SNPs with suboptimal calling quality (https://www.illumina.com/content/dam/illumina/gcs/assembled-assets/marketing-literature/gentrain-tech-note-m-gl-01258/gentrain-tech-note-m-gl-01258.pdf) (Zhao *et al*. 2018). To observe the full range of potential predictions, no additional quality control was applied to the samples in our analysis; however, users may additionally upload a PLINK v1.9 (RRID: SCR_001757) (Purcell *et al*. 2007) bim format file or PLINK v2.3 (Chang *et al*. 2015) pvar format file to limit variants to those that passed quality control detailed elsewhere (Vitale *et al*. 2025). Furthermore, users are required to specify either a gene name or chromosome, along with start and stop positions in base pairs, defining the region of interest. All graphical representations and positional references in this paper adhere to the Genome Reference Consortium Human Build 38 (hg38) (Guo *et al*. 2017).

### 2.2 Samples for model development

All GP2 cohorts involved in model creation and evaluation can be searched on GP2's Cohort Dashboard by their study abbreviations (https://gp2.org/cohort-dashboard-advanced/). Table S1 presents the manually curated set of 184 samples with 156 PD cases and 28 controls that were used in training preliminary models for each CNV type. The preliminary training set comprises 70 deletions (Table S1a), 29 duplications (Table S1b), and 85 samples lacking visible CNVs in *PRKN* (Table S1c).

Our initial training set was iteratively expanded by incorporating both confirmed carriers and rejected candidates following manual review of CNV-Finder’s predictions across the cohorts listed in Table S2. These samples encompass a broad spectrum of phenotypes and span 11 ancestries to diversify the training sets. The process by which samples were incorporated into the preliminary, updated, and final models for each CNV type is summarized in Fig. S1 with the annotation procedure underlying these decisions detailed in Section 2.3.

Expert annotations on samples from the Coriell Institute for Medical Research (CORIELL), detailed in Table S3, were withheld during preliminary and updated model training. This served to assess performance improvements between the two models and provide an external benchmark whose ground-truth labels were established entirely independently of CNV-Finder predictions, eliminating any influence of the iterative annotation process on this evaluation. CORIELL samples were subsequently incorporated into our largest, final training set.

### 2.3 Human-in-the-loop annotation process

Visual assessment was performed by a team of 13 research scientists with expertise in neurodegenerative disease genetics, who annotated the initial dataset of *PRKN* carriers, including the held-out CORIELL samples. This sample set was annotated in Illumina’s GenomeStudio (https://www.illumina.com/products/by-type/informatics-products/microarray-software/genomestudio.html) prior to any CNV-Finder model development, such that no model predictions were visible during its assessment, ensuring that this benchmark was established entirely independently of model output.

A subset of 7 annotators conducted the subsequent rounds of iterative review used to expand the training sets. Samples were reviewed through the CNV-Finder web application. Similar to GenomeStudio, it displayed LRR and BAF plotted against chromosomal position for the region of interest. Each sample was assigned one of three labels: “Yes” (a visible CNV of the relevant type), “No” (no visible CNV), or “Maybe.” The “Maybe” category was used deliberately to prevent miscategorization of ambiguous samples such as those exhibiting characteristic LRR patterns but sparse BAF data, or potential artifacts. These samples were excluded from the training sets rather than forced into a binary class.

For the initial *PRKN* training set, short-read WGS was used to confirm the absence of structural variants in samples labeled “No.” The Accelerating Medicines Partnership program for Parkinson’s disease (AMP-PD) short-read WGS data collection has been previously outlined by Iwaki *et al*. (2021) with additional details on SNV calling by Billingsley *et al*. (2023). All samples were annotated independently and cases in which annotators disagreed were resolved through consensus discussion. Any sample for which reviewers could not reach a decision was excluded, minimizing the risk of noise in the training data. Following manual review of model predictions, both confirmed carriers and rejected candidates with prediction values above 0.8 were incorporated into the expanded training sets.

### 2.4 Samples for evaluation

To benchmark model confidence, resource utilization, and the degree to which manual validation effort could be reduced, CNV-Finder was applied to *PRKN* deletions and duplications across 1 05 936 GP2 samples with available array data that were not utilized in model development. Performance of our final models were further evaluated using multi-modal and extended datasets from additional studies. Samples from the Parkinson’s Families Project (PFP) (Towns *et al*. 2024) and Parkinson’s Disease in Southern Africa (STELLENBOS) (Keyser *et al*. 2010, Haylett *et al*. 2012, Braun *et al*. 2025), cohorts of roughly the same size totalling 2971 samples described in Table S4, were used for prediction comparisons against one of the most widely used array-based CNV callers, PennCNV (Wang *et al*. 2007), in *PRKN* deletions and duplications. Both methods were provided identical regional input to enable direct comparison of true positive rates. These cohorts were additionally used for Multiplex Ligation-dependent Probe Amplification (MLPA) validation of *PRKN* and *SNCA*. Parkinson’s Progression Markers Initiative (PPMI) samples highlighted in Table S5 enabled short and long-read WGS validation of the three genes involved in model development. Finally, a subset of 506 samples from the (MDGAP-DEMENTIA) and REasons for Geographic and Racial Differences in Stroke (REGARDS) studies in Table S6 supported an in-depth exploration of the 17q21.31 region encompassing *MAPT*, particularly within dementia-related phenotypes.

### 2.5 Long-read whole genome sequencing

As detailed at dx.doi.org/10.17504/protocols.io.x54v9py8qg3e/v1, DNA was extracted from 1 mL of frozen whole blood using the KingFisher Apex instrument and an adapted version of the PacBio Nanobind HT HMW DNA Extraction protocol with the Nanobind HT 1 mL Blood Kit. High molecular weight DNA was size-selected with the PacBio Short Read Eliminator Kit to remove fragments up to 25 kilobases (kb), then sheared to a size of 30 kb using the Megaruptor 3 and Diagenode Shearing Kit. DNA libraries were prepared with the Oxford Nanopore Technologies (ONT) SQK-LSK114 Ligation Sequencing Kit and sequenced on PromethION R10 flow cells (FLO-PRO114M) for 72 hours.

Raw signal data (Fast5 files) were generated using ONT MinKNOW v22.10.7 (https://nanoporetech.com/about-us/news/introducing-new-minknow-app). Super accuracy basecalling was performed with ONT Dorado v0.6.0 (RRID: SCR_025883). Quality-filtered Fastq files were aligned to the hg38 reference genome using Winnowmap (RRID: SCR_025349). SAM files were converted, sorted, and indexed into final BAM files using SAMtools v1.20 (RRID: SCR_002105) (Li *et al*. 2009). Structural variants (SVs) were detected and genotyped using Sniffles2 v2.3 (RRID: SCR_017619, https://github.com/fritzsedlazeck/Sniffles) (Smolka *et al*. 2024).

## 3 Algorithm

### 3.1 Model framework

Our pipeline employs a recurrent neural network known as Long Short-term Memory (LSTM) (Hochreiter and Schmidhuber 1997), known for its proficiency in capturing long-term dependencies within sequential data. We stack three LSTM layers, each passing its sequential representations to the next, forming a deeper network that learns multiple levels of abstraction for improved feature extraction. A hard sigmoid activation function was chosen for its decreased computational cost and success with deep learning binary classification tasks. Therefore, our model outputs predicted values between 0 and 1 which correspond to the likelihood of a CNV for that sample in the region of interest (Fig. S2).

We apply overlapping sliding windows to the signal intensity files during data preparation for our model. To determine window sizes for each gene or interval of interest, we utilize customizable variables for “split” and “total window counts” within the region. For instance, if a model is trained with a split of 5 and a total window count of 50, this signifies that a gene like *PRKN* is divided into 5 equal segments, each spanning 2 76 049 base pairs without any overlap. Subsequently, 50 windows are computed to overlap these segments by 22 534 bases as shown in Fig. S3. Similarly, in *MAPT*, the 5 equal intervals are 26 790 base pairs long with windows overlapping by 2168 bases. This approach creates proportional windows to span any gene regardless of its size, without the need for padding with artificial bases to create sequences of equal lengths. Furthermore, it enables the application of a single model to all regions prepared with the same split and window counts. We also include a modifiable buffer that can be added to both sides of a region of interest before window calculation. In our analysis, we incorporated a default 250 kb buffer to facilitate the exploration of variant behavior surrounding the regions of interest. We later adjusted this metric to 10 megabases (Mb) for the analysis of *SNCA*, to better capture the scale of large SVs found in this risk locus.

**Figure 1:**
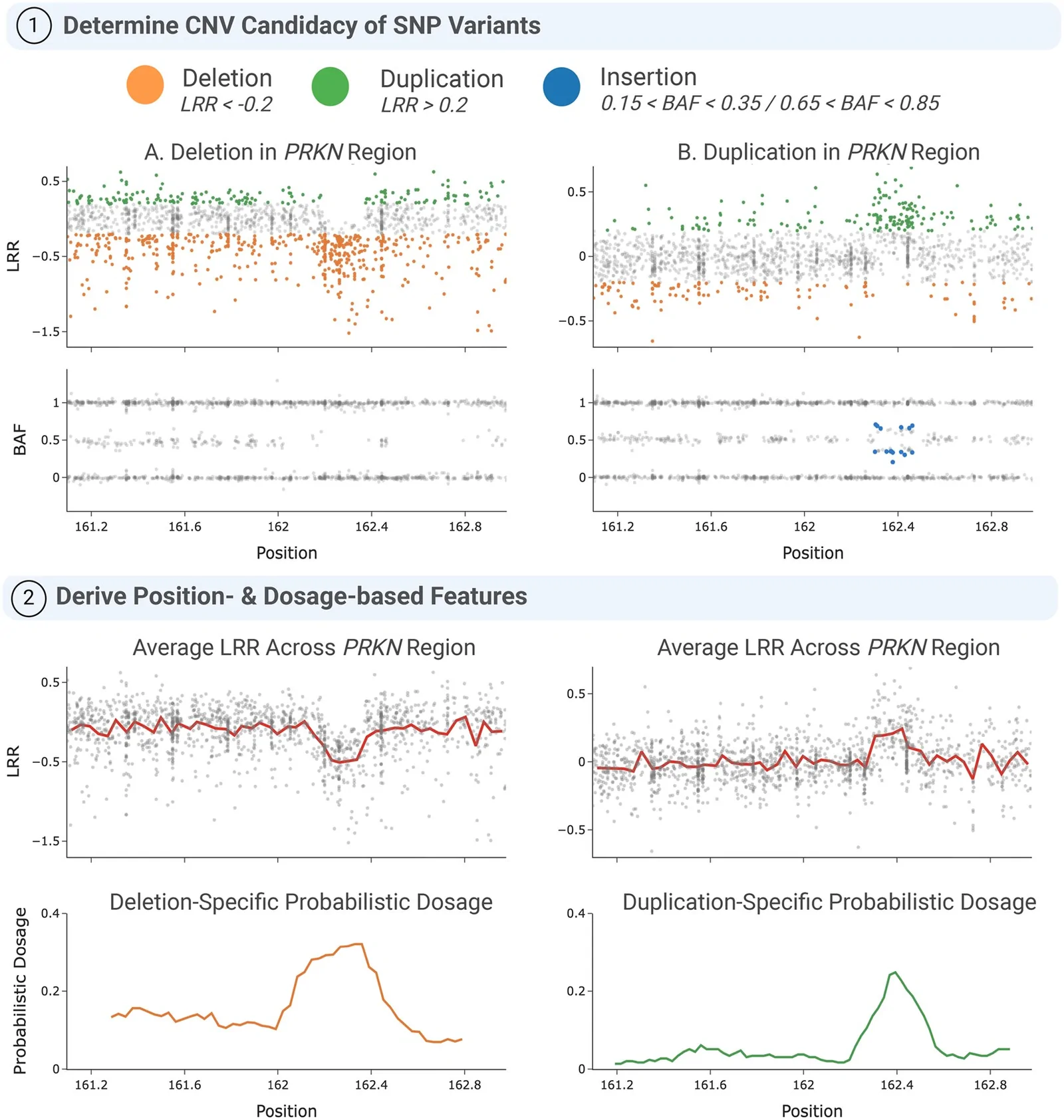
Feature creation to capture deletions (A) and duplications (B). (1) Illumina-based thresholds are applied to SNPs to determine if their LRR and BAF values fall into the proper ranges to be considered deletions and duplications. (2) Features in Table S7 are calculated on the variants within these specified CNV ranges (CNV candidates).

Features summarized in Table S7 are aggregated within and across each window to capture trends such as visible positional shifts in LRR or BAF and probabilistic dosage spikes. These spikes are depicted in Fig. 1 by LRR or BAF against chromosome position (in base pairs) for *PRKN* deletions (Column A) and duplications (Column B). SNP variants in this figure, color-coded for visibility, are deemed “CNV candidates” if they fall within specific ranges recommended by the array manufacturer:

- Deletions: LRR < -0.2
- Duplications: LRR > 0.2
- Insertions: 0.15 < BAF < 0.35 or 0.65 < BAF < 0.85

To comprehensively capture the mid-range of BAFs, we incorporate additional features encompassing BAF values from 0.15 to 0.85, inclusive. This broader range of frequencies is crucial for capturing characteristics such as the diverging branching pattern consistently observed in duplications of varying size and BAF values (Blauwendraat *et al*. 2021). Most model features are only derived from the CNV candidates, aiming to enhance precision and reduce computational cost by subsetting the extensive pool of SNPs within each region.

A five-fold cross-validation was performed on the training set to ascertain optimal window sizes and feature combinations, listed in Table S7, for predicting each CNV type. A split of 5, sliding window of 50, and the 4th feature combination, earned an average Area Under the Curve (AUC) (Table S8) of 0.93 for our deletion model. For the duplication model, an average AUC of 0.92 was obtained with a split of 10, sliding window of 70, and the 6th feature combination. Therefore, if you choose to utilize our existing pre-trained models for a cohort of size N samples, the pipeline will reshape the model-ready input files to the necessary dimensions of [N, 50, 13] and [N, 70, 11] for the deletion and duplication models, respectively.

### 3.2 Semi-automated integration with visualization

The final step of CNV-Finder prepares app-compatible files for visualization within our interactive web application. This interface enables researchers to review predictions and filter displayed samples by features such as model prediction values in order to explore or confirm results. Users may examine results by selecting from options such as “Yes,” “Maybe,” or “No,” which are used to record chosen sample IDs, their respective regions of interest, and the type of CNV under evaluation. Logs generated by the application can subsequently serve as inputs to downstream analyses drawing conclusions about CNV types and sample phenotypes, or for training a new model.

## 4 Implementation

CNV-Finder was developed using over 11 000 samples and trained through an iterative framework that produced preliminary, updated, and final models, all sharing the same architecture but built on progressively larger training sets. Large-scale benchmarking of the final models were performed at the *PRKN* locus in 1 05 936 previously unseen GP2 samples. Table 1 presents nominated and visually confirmed predictions stratified by 10-percentile prediction score intervals, demonstrating how model accuracy increases with model confidence and helping users identify the optimal threshold for maximizing the efficiency of their manual validation efforts. Even when low-probability predictions were included, only 3% of the total cohort were nominated as potential deletion carriers and less than 1% as potential duplication carriers. At the highest confidence tier (prediction scores ≥ 0.9), the model achieved its greatest precision, confirming 743 of 818 nominated deletions (91%) and 381 of 429 nominated duplications (89%).

**Table 1:** *PRKN* CNV nominations and visual confirmation rates across GP2 samples stratified by prediction value range.

Prediction value range	Deletions		Duplications	
	Nominated	Confirmed	Nominated	Confirmed
0.90–1.00	818 (0.77%)	743 (90.83%)	429 (0.40%)	381 (88.81%)
0.80–0.89	145 (0.14%)	94 (64.83%)	13 (0.01%)	4 (30.77%)
0.70–0.79	222 (0.21%)	71 (31.98%)	6 (0.01%)	0 (0%)
0.60–0.69	579 (0.55%)	33 (5.70%)	9 (0.01%)	1 (11.11%)
0.50–0.59	1709 (1.61%)	9 (0.53%)	13 (0.01%)	3 (23.08%)

Nominated values represent the count and percentage of total samples (*n* = 105 936) nominated at each threshold.

The pipeline supports parallel processing across cohorts and loci, enabling efficient multi-core implementation. For a cohort of around 1500 samples, the most computationally intensive steps of data processing and feature aggregation generated ∼30 MB machine learning-ready files in under 20 minutes using 4 CPUs. Training the largest final duplication model (*n* = 594) produced a model file in 31 seconds, available in either Keras (1 MB) or ONNX (345 KB) format. Testing the pre-trained model required only 9 seconds with a single CPU. ONNX (https://onnx.ai/) formats enable interoperability by allowing users to reach equivalent prediction values across different programming environments without requiring the original training framework.

## 5 Discussion

### 5.1 Visual validation to assess iterative training sets

Even with its smallest training set, CNV-Finder correctly identified 97% of the held-out, expert-annotated CORIELL deletions and 84% of the duplications in *PRKN* with predicted values above 0.5 (Table 2). These CORIELL annotations were established through independent expert visual review prior to any model development, ensuring that this benchmark was entirely free from the influence of model predictions. Subsequent sample additions to the training set reduced false positives of the deletion model and decreased false negatives of the duplication model, while maintaining high rates of true positives in the updated model. This improvement is most likely attributable to the inclusion of both confirmed carriers and rejected candidates in the expanded training set, exposing the model to samples that looked like CNVs to the model but were rejected by experts. This counteracts any chance of the human-in-the-loop process only reinforcing what the model already does well. Consequently, this led to increases in the AUC metrics, from 0.92 to 0.99 for deletions and 0.91 to 0.92 for duplications.

**Table 2:** Preliminary and updated model results for known *PRKN* CNVs in held-out CORIELL PD cases.

	Deletions		Duplications	
	Preliminary model	Updated model	Preliminary model	Updated model
True Positives	146	147	32	33
True Negatives	3607	4103	4070	4058
False Positives	537	41	74	86
False Negatives	4	3	6	5
Brier Score	0.114	0.055	0.017	0.025
Sensitivity	0.97	0.98	0.84	0.87
Specificity	0.87	0.99	0.98	0.98
AUC	0.92	0.99	0.91	0.92

Visually confirmed and rejected samples with predicted values above 0.8 were fed back into the updated model’s training set to create the final training sets for our models.

Model refinement also substantially reduced the number of high-confidence predictions requiring manual review. In CORIELL, preliminary models identified 925 deletion and 333 duplication candidates with scores over 0.8, whereas updated models reduced these to 100 and 126, respectively. Among high-confidence predictions, most deletion candidates (96/100) and a subset of duplication candidates (53/126; all over 0.9 probability) were confirmed and incorporated into the final training sets. Updated models also reduced Brier scores and captured diverse patterns. This includes homozygous deletions and noisy duplications, further improving overall performance and generalizability (Table S8; Fig. S4).

### 5.2 Final model performance

#### 5.2.1 Comparison to existing pipelines

Using all samples from the STELLENBOS and PFP cohorts, we compared the ability of PennCNV and CNV-Finder to predict both intronic and exonic *PRKN* CNVs, with performance assessed through expert visual confirmation and MLPA validation. Given that elevated false positive rates are a well-recognized challenge in array-based CNV detection, we were particularly interested in each method’s precision. Table 3 compares nomination and visual confirmation rates for all *PRKN* deletions and duplications across both cohorts. CNV-Finder nominated substantially fewer samples at prediction values > 0.5 than PennCNV while achieving higher confirmation rates, nominating 80 deletion carriers with an 84% confirmation rate compared to PennCNV’s 506 nominations at only 16%, and nominating 17 duplication carriers with a 100% confirmation rate compared to PennCNV’s 58 nominations at 36%. These results demonstrate that CNV-Finder substantially reduces the burden of manual review while maintaining sensitivity for MLPA-confirmed exonic CNVs, offering a precise and efficient approach to array-based CNV detection.

**Table 3:** Comparison of *PRKN* CNV nominations and confirmation rates between PennCNV and CNV-Finder across PFP (*n* = 1454) and STELLENBOS (*n* = 1517) cohorts for (A) deletion and (B) duplication results.

Cohort	Modality	PennCNV		CNV-Finder	
		Nominated	Confirmed	Nominated	Confirmed
(A) Deletions					
STELLENBOS	Visual	66	36 (55%)	35	30 (86%)
	MLPA	11	8 (73%)	9	8 (89%)
PFP	Visual	440	45 (10%)	45	37 (82%)
	MLPA	270	19 (7%)	22	18 (82%)
Total	Visual	506	81 (16%)	80	67 (84%)
	MLPA	281	27 (10%)	31	26 (84%)
(B) Duplications					
STELLENBOS	Visual	32	7 (22%)	5	5 (100%)
	MLPA	2	0 (0%)	1	1 (100%)
PFP	Visual	26	14 (54%)	12	12 (100%)
	MLPA	7	0 (0%)	5	5 (100%)
Total	Visual	58	21 (36%)	17	17 (100%)
	MLPA	9	0 (0%)	6	6 (100%)

Confirmed samples represent visually confirmed (Visual) or MLPA-validated (MLPA) candidates, with confirmation rates shown in parentheses.

MLPA currently serves as the gold standard for confirming deletions and duplications in exonic regions that are crucial to determining disease relevance. Therefore it is imperative that we validate our predictions with this modality. All samples with known mutations that could lead to false positive MLPA results were excluded. Although both methods can also detect intronic CNVs, these SVs were excluded from our comparisons to match the functionality of MLPA. PennCNV nominations were retained only if the reported CNV positions overlapped with at least one of *PRKN*'s 12 exons. Table 3 compares MLPA-confirmed *PRKN* CNVs between the two methods across both cohorts. For deletions, CNV-Finder nominated substantially fewer candidates than PennCNV (31 versus 281) while achieving a markedly higher MLPA confirmation rate (84% versus 10%). For duplications, PennCNV failed to confirm any of its 9 nominated candidates (0%), whereas CNV-Finder confirmed all 6 of its nominations (100%). These results highlight CNV-Finder’s increased precision in identifying true exonic *PRKN* CNV carriers.

Although both cohorts are part of GP2 and were genotyped on the same array, their independence allows for assessment of performance stability across cohort-specific effects. Compared to the HMM probabilistic framework employed by PennCNV, LSTMs can selectively retain relevant signal over longer ranges while suppressing noise, making them well-suited to the sequentially-derived LRR and BAF signals, which may not conform to the generic state parameters specified by the HMM. While the HMM relies on its emission and transition distributions being correctly specified in advance, CNV-Finder instead estimates the relationship between signal and CNV state directly from labeled data, allowing it to capture both the classic CNV patterns an HMM models and the cohort-specific noise structure that a fixed parametric model may not anticipate. This flexibility in distinguishing true CNV signatures from the noise common in array data likely contributes to the more consistent performance of CNV-Finder across the two cohorts, compared to PennCNV’s substantial variation in the number of STELLENBOS and PFP nominations. An HMM may benefit more than CNV-Finder from variant quality control that we did not perform prior to our analysis. If such filtering does not substantially reduce probe density over the region of interest, it could bring noisier cohorts closer to the distributions the HMM model was specified to capture.

Table S9 further contextualizes these findings by examining the size distribution of confirmed CNVs detected by each method. Both callers captured variants across a broad size range from less than 10 kb to over 500 kb. Despite nominating substantially fewer candidates overall, CNV-Finder missed a small number of sub-10 kb variants that were nominated by PennCNV, indicating reduced sensitivity at the lower end of the size spectrum. This is an expected constraint of array-based CNV detection, where probe density fundamentally limits resolution for smaller variants. It is worth noting that within CNV-Finder’s gene-level framework, detectability is governed less by absolute CNV size and more by the relative size to the target region. Therefore, a CNV spanning a substantial proportion of the gene’s probe coverage is likely to be captured regardless of its absolute size in base pairs.

#### 5.2.2 MLPA validation on *PRKN* and *SNCA*

Examining the MLPA validation results more closely, we analyzed PFP and STELLENBOS samples in *PRKN*, our training set’s most prominent gene, and in *SNCA*, which was not included in model development. Table 4 shows our full MLPA validation results with additional performance listed for samples that are visible to our experts in LRR or BAF vs. position plots. Although validation on the full set shows strong performance with AUCs ranging from 0.83 to approximately 1, accuracy is even higher for visually identifiable samples, ranging from 0.92 to 1 due to the models’ dependence on features derived from these plots. Figure S5 illustrates the concordance between NBA-based prediction values and visible plot features in *PRKN*.

**Table 4:** Summary of model predictions on combined MLPA data from PFP and STELLENBOS cohorts (*n* = 1234).

	PRKN deletions	PRKN duplications	SNCA duplications*
True Positives	26	6	7
True Negatives	1201	1226	1188
False Positives	5	0	39
False Negatives	2	2	0
Brier Score	0.036	0.003	0.027
Sensitivity	0.929	0.75	1
Specificity	0.996	1	0.968
AUC	0.979	0.826	0.996
AUC for Visible Samples	1	0.922	0.996

Asterisks denotes that the final duplication model was used to make predictions on *SNCA*; however, both duplications and triplications were identified.

**Figure 2:**
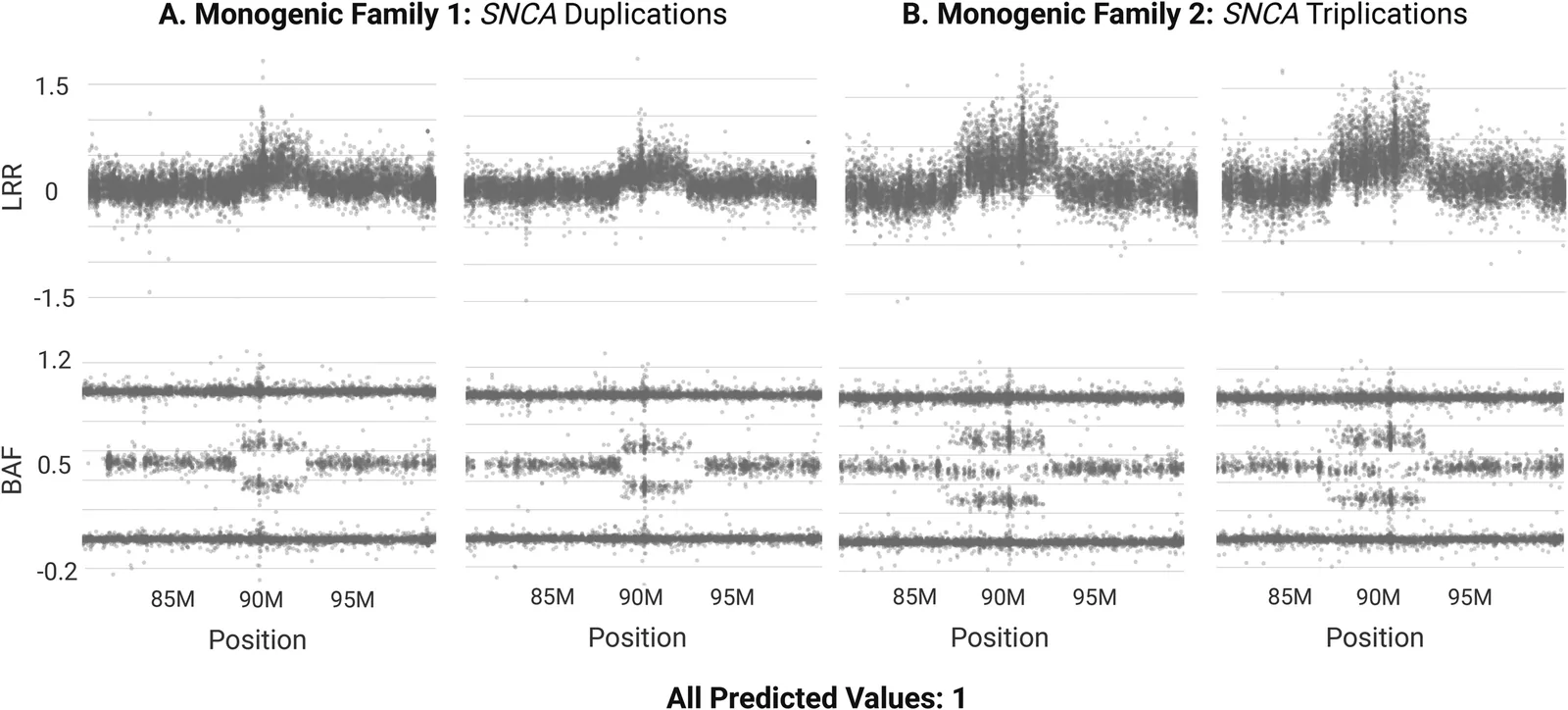
MLPA-validated *SNCA* multiplications from two monogenic families with their respective prediction values determined by CNV-Finder’s duplication model.

We further confirmed that our duplication model captures both *SNCA* duplications and triplications with equally strong prediction values. Figure 2 features two monogenic families with duplications and triplications, demonstrating the generalizability of our models, despite the lack of any *SNCA* or triplication examples in the training sets. The lower frequency and clinical significance of CNVs in *SNCA* makes it crucial that our pipeline can find all positive samples without prior exposure to this gene. Moreover, the model maintained high specificity, assigning prediction values above 0.5 to less than 40 out of 1234 samples, thereby limiting user review to only 3% of the dataset.

#### 5.2.3 Short- and long-read sequencing validations

We used both Illumina short-read and ONT long-read WGS to validate predictions in the three genes incorporated in our model development. Long-read sequencing can identify distinct CNVs beyond the resolution of short reads and arrays, making it difficult to define false negatives in relation to array-based predictions (Lavrichenko *et al*. 2021). In contrast, short-read data is constrained by relatively high rates of false positives, also complicating the estimation of false negatives and potentially skewing true positive rates with respect to CNV-Finder’s results (Pirooznia *et al*. 2015).

**Figure 3:**
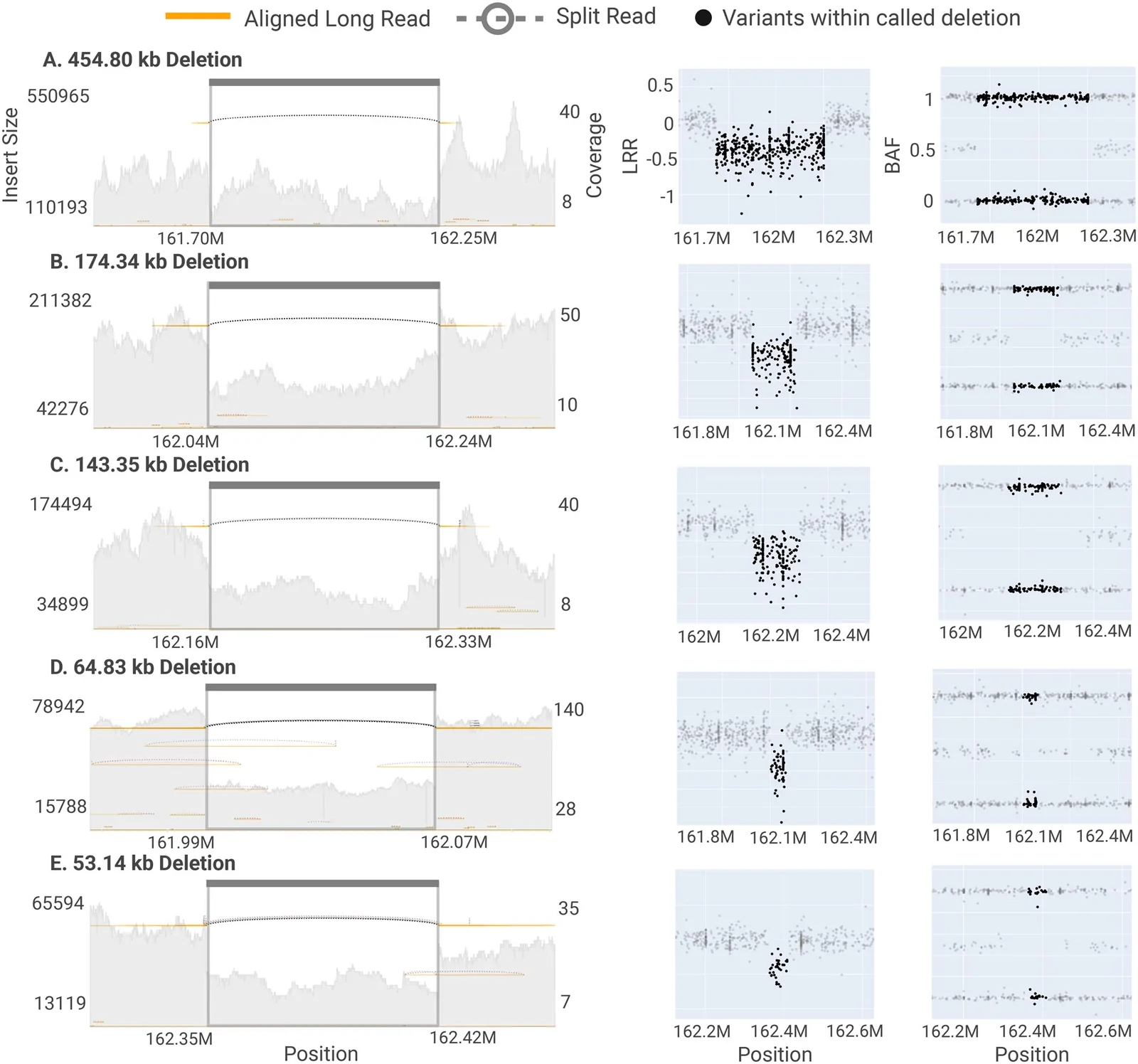
Long-read WGS validation of predicted deletions in *PRKN*. Four samples (A, B, C, E) received prediction values of 1 and one sample (D) received 0.93.

Our focus was on utilizing ONT long-read WGS data to validate samples with prediction values exceeding 0.9, to ensure that the strongest candidates from our models were not false positives. Figure 3 showcases five samples with predicted and confirmed *PRKN* deletions by long-read sequencing. These deletions vary in size, ranging from approximately 53 kb to 455 kb.

**Figure 4:**
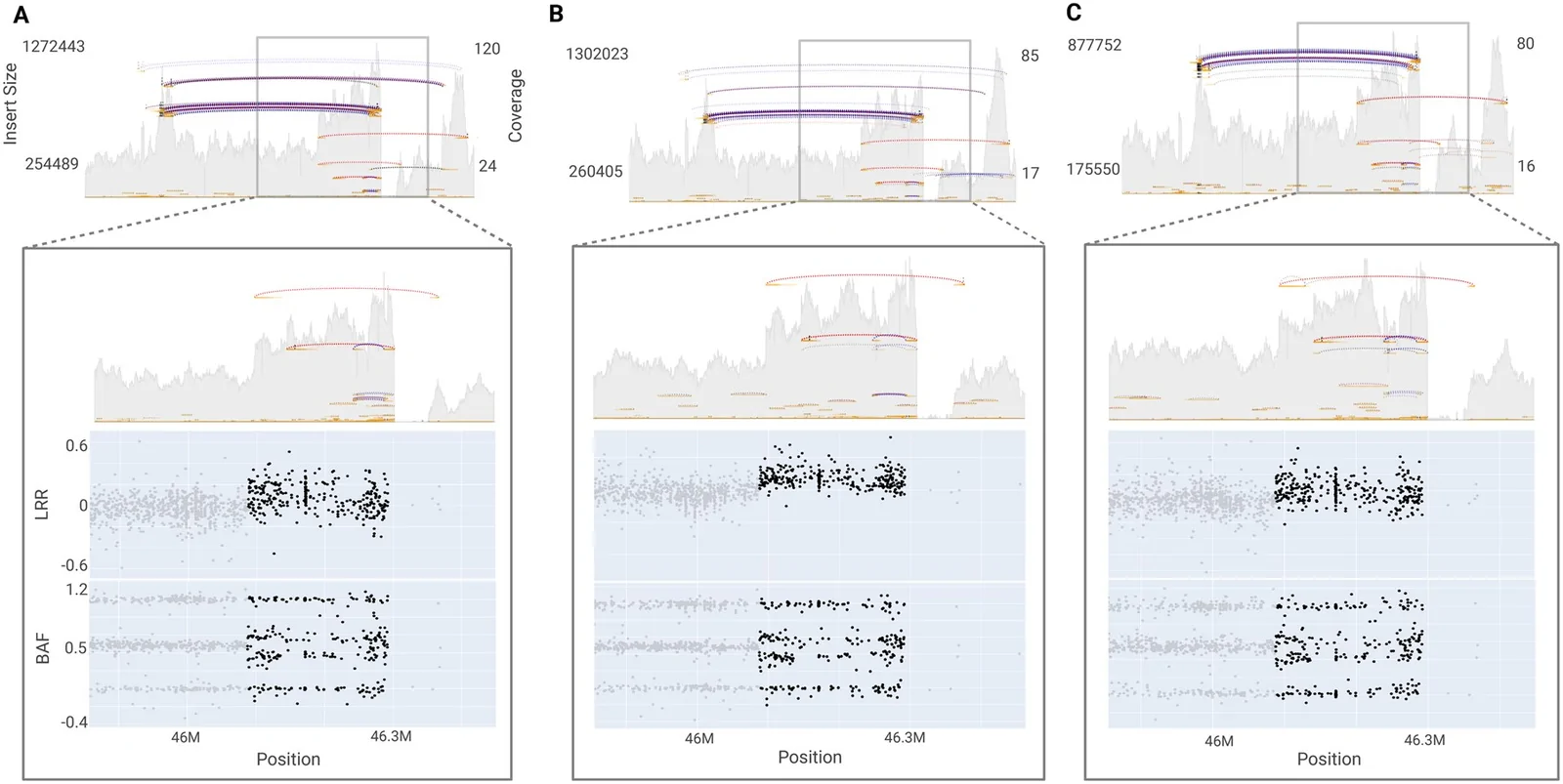
Long-read WGS validation of predicted duplications near *MAPT*. All three samples received prediction values of 1.

Additionally, Fig. 4 features three duplications near *MAPT* validated by long-read sequencing. As previously mentioned, a 250 kb buffer surrounding the gene interval was employed in our analyses, enabling us to capture these duplications in the regions overlapping *MAPT* and *KANSL1*. Despite variations in the exact shape and variant noise levels, all validated CNVs received predicted values of 1.0 from their respective models, with only one predicted deletion obtaining a value of 0.93.

**Figure 5:**
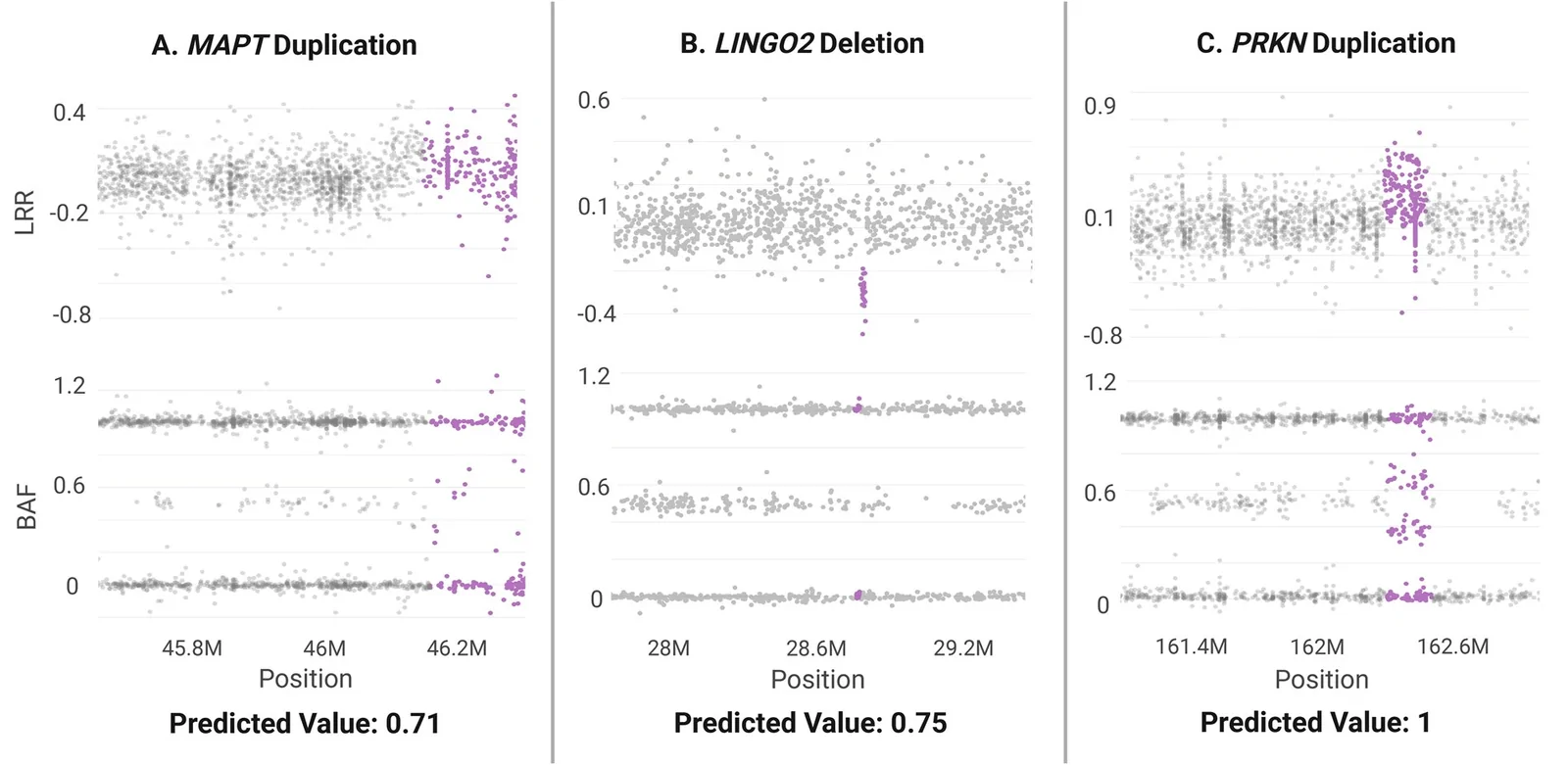
Short-read WGS validation of model results with varied prediction values.

We incorporated short-read sequencing of an additional 337 samples to further explore predictions with values exceeding 0.5. Figure 5 presents CNVs confirmed by short-read WGS across a range of model prediction scores, with pink-highlighted regions indicating deletions or duplications identified by this modality. Model prediction values and visual clarity of the confirmed CNVs increase from left to right in the figure. Notably, *LINGO2* features the smallest confirmed deletion in the group, with a width of only about 27 kb.

Table 5 breaks down the Positive Predictive Value (PPV) values for long-read and short-read WGS comparisons. PPV scores on samples with predicted values over 0.9 were high for long-read validation, demonstrating a score of 1 for *PRKN* deletions (*LINGO2* deletions were excluded due to insufficient samples) and 1 for duplications near *MAPT* (*PRKN* duplications were excluded due to insufficient samples). For short-read comparisons on samples with predicted values over 0.5, PPV averaged 0.95 for the final deletion model among *PRKN* and *LINGO2* while the final duplication model achieved an average of 0.90 for *PRKN* and *MAPT*. The leniency in *MAPT*’s false positive rate is attributed to the training set selections described in the following section.

**Table 5:** Summary of Positive Predictive Values (PPV) for final model predictions above 0.9 on PPMI long-read WGS data and predictions above 0.5 on short-read WGS data.

	Deletions		Duplications	
	*PRKN*	*LINGO2*	*PRKN*	*MAPT*
Long-read WGS				
True Positives	6	0	0	3
False Positives	0	0	0	0
PPV	1	**–**	**–**	1
Short-read WGS				
True Positives	16	4	3	53
False Positives	2	0	0	14
Average PPV	0.95		0.9	

### 5.2 Duplications within the 17q21.31 region

**Figure 6:**
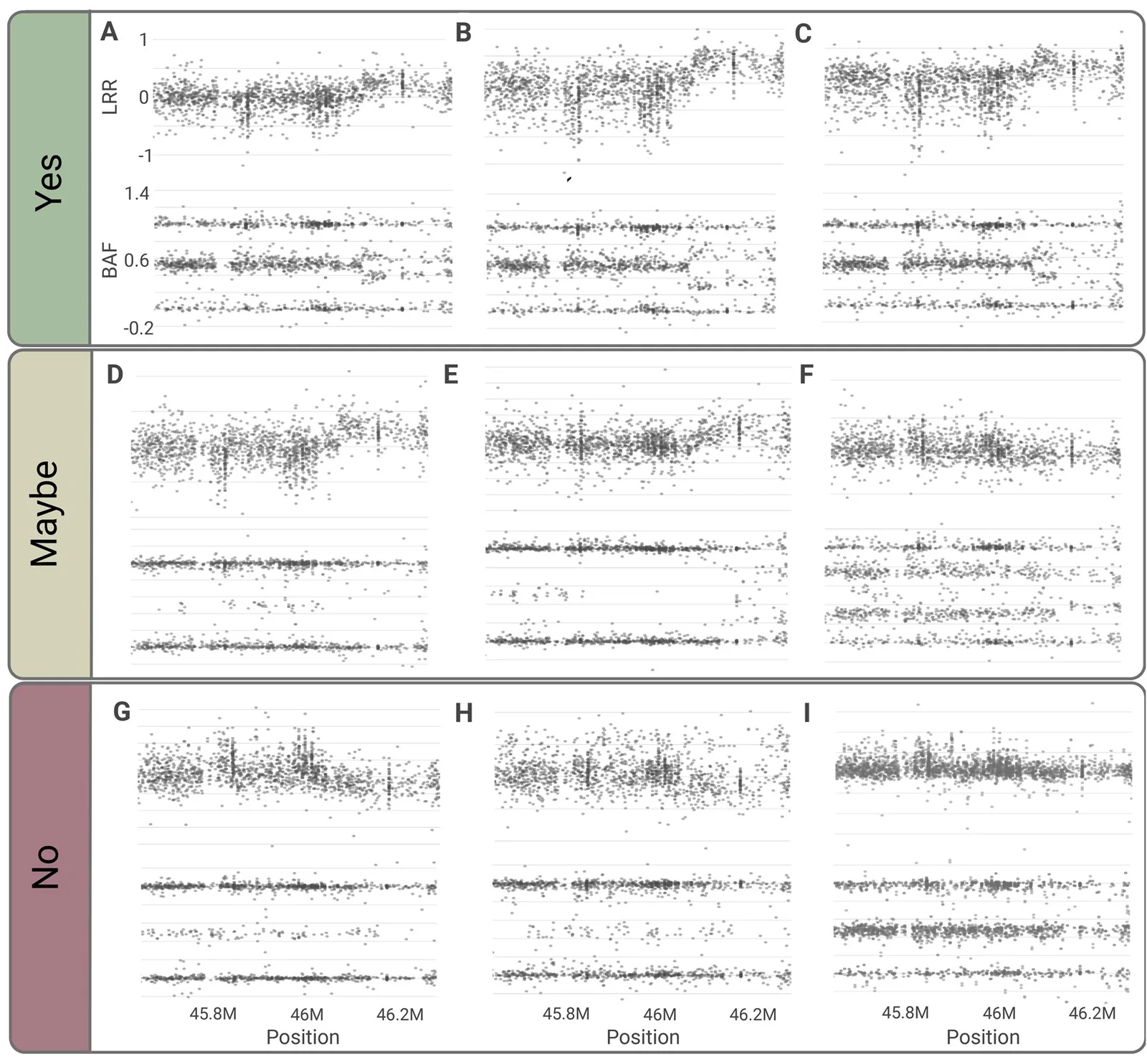
Examples of classification used on duplication near MAPT during visual confirmation.

Further exploration of the *MAPT* region highlights the crucial role of contextual understanding and human proficiency in enhancing the precision of CNV identification. Deliberate consideration was given to the classifications of “Yes,” “Maybe,” and “No” within the CNV-Finder application when selecting samples to add to the final training set. Figure 6 showcases examples corresponding to each selection category. Samples D and E in this figure were marked as “Maybe” to avoid mislabeling them as negative samples with no CNVs. Typical duplication patterns are discernible in their LRR versus position plots while the BAF vs. position plots lacked sufficient data. Additionally, potential artifacts like Sample F were marked as “Maybe” to prevent inclusion in the training sets. The duplication pattern highlighted in the “Yes” column, consistently appearing at locations around 461 kb–463 kb on the 17th chromosome near *MAPT* and within the *KANSL1* region, exhibited the highest prevalence among the inspected CNVs across our three genes of interest. Across all cohorts reviewed for model development, a total of 737 samples exhibited visible duplications, while 414 samples showed no or limited evidence of duplication in the *MAPT* region, resulting in the annotation of 1151 samples with “Yes” or “No” for this variant.

As previously mentioned, the 17q21.31 region which encompasses the *MAPT* gene, is made complex by an H1 haplotype and inverted H2 haplotype. To better analyze this region and distinguish between haplotypes, we examined the H1/H2 tagging SNP rs8070723 across the 1151 annotated samples, revealing a Jaccard similarity score (Table S8) of 0.94 between H2-carrier status (H1H2 + H2H2) and the presence of an obvious duplication in the region (“Yes” classification). A logistic regression analysis was conducted on an 80:20 partition of these samples, ensuring consistency in the distributions of population ancestries across both data subsets. This resulted in a 97% accuracy and an AUC of 0.95 when predicting the existence of a duplication in this region using only H2-carrier status as the feature. There were only 6 false positives and 0 false negatives in the test set comprising 146 samples with the observed duplication and 85 samples without.

Furthermore, to better examine this variant in H2 non-carriers (H1H1), we applied our final duplication model to AD and dementia cases in the MDGAP-DEMENTIA and REGARDS cohorts. Among the 506 cases included in these cohorts, H2 non-carriers constitute 64%. Upon visual inspection of all samples with predicted values over 0.5, 38 instances were found to meet the criteria typically associated with a “Yes” designation in Fig. 6. These instances encompass a variety of NDDs, including cases of AD, FTD, PSP, DLB, MSA, CBD/CBS, Undetermined-Dementia, and other tauopathies; however, all are H2-carriers. Conversely, out of the 75 samples annotated as “Maybe” due to sparse BAF vs. chromosome position plots resembling those seen in plots D and E of Fig. 6, all are H2 non-carriers (H1H1). One sample reported as a “No Call” for SNP rs8070723 was categorized as “No.” Had the duplication model not been trained with careful consideration for the expansion of both binary classes (CNV exists versus CNV does not exist), the final model would not have assigned high probabilities to H2 non-carriers, thus neglecting their flagging for human review. This highlights the significance of contextual interpretation by researchers and the additional layer of insight their expertise provides, particularly when array-based plots may not fully reflect all pertinent information.

### 5.2 Utility

CNV-Finder facilitates streamlined and comprehensive analysis of deletions and duplications, capable of handling sample sizes necessary for capturing rare SVs and enhancing the statistical power of disease associations. This pipeline operates independently of outputs from any supplementary CNV prediction or imaging software, harnessing the extensive advantages of contemporary deep learning models with a simple user interface and integrated data visualization. Its optional semi-automated functionality allows for the integration of expert validation, a process traditionally essential for confirming predicted candidates from standard array-based callers. Human expertise proves invaluable, particularly in complex regions like 17q21.31, where additional context is needed for accurate interpretation of detected variants or for navigating artifacts that may mimic structural variant features. Models trained with user visual confirmation have demonstrated enhanced detection capabilities for noisy and small CNVs, concurrently reducing false positive rates.

### 5.2 Limitations and next steps

It is noteworthy that while human validation within CNV-Finder’s semi-automated approach has enhanced model performance in our analyses due to careful consideration and benchmarking, it can also introduce inconsistencies and biases to predictions if new models were trained. The balance and representation of each class (samples with CNVs versus samples without CNVs) in the training set may be influenced by selections in a manner detrimental to prediction accuracy. Additionally, newly added positive examples are restricted to CNVs nominated by the model. Therefore, the expanded training data may be enriched for CNV signatures the model can already detect, potentially limiting improvement on signatures it currently misses. Users are encouraged to first apply CNV-Finder’s pre-trained final models before considering training a new model from scratch as these have indicated sufficient generalizability that has not significantly hindered their performance.

In addition, our use cases have been confined to regional explorations with binary outputs from a fixed-length, sequence-to-vector LSTM model. This decision was made to facilitate rapid predictions on gene regions with established significance or interest demonstrated in the literature. Future iterations of the model will leverage LSTMs’ capacity to generate sequences of model prediction values. Although this adjustment may increase computational cost, it could enable users to apply the model across the entire genome, with outputs indicating more specific regions of elevated probability for each CNV type. We may also introduce new features that provide additional context to our predictions. These may involve a more selective inclusion of array probes in the input files, an addition of region-specific SNPs such as the *MAPT* H1/H2 tagging variant, or more granular classifications of CNV types, as in the case of *SNCA* triplications and duplications.

Future studies will need to investigate the performance across a wider spectrum of genotyping arrays, which may exhibit varying levels of call quality and coverage of genetic variants. Reduced probe density is expected to lower performance through the resolution limits of array-based detection itself, not through any model-specific effect. Beyond this, the nature of our model architecture and its use of sliding windows, make it possible to adapt our models to features from short-read WGS, such as depth-of-coverage, to potentially improve predictions through the enhancements in call quality from this modality.

Finally, as previously reported, the 17q21.31 region challenges the accuracy of short- and long-read SV calling (Billingsley *et al*. 2023). Conventional variant calling algorithms often struggle to precisely determine the position ranges of potential CNVs within this genomic region. For instance, Sniffles2 (Smolka *et al*. 2024), a widely-used open-source tool for analyzing long-read SVs, identified duplications in our PPMI samples proximal to *MAPT*, spanning positions 46 135 410 to 46 292 247 on chromosome 17, across both short-read and long-read sequencing data. However, upon visual examination of the three assessed samples (referenced in Fig. 4) alongside SAMtools plots derived from long-read sequencing data, it became apparent that the start of the CNV likely occurred earlier than reported. Moreover, the loss of sequencing coverage immediately following the duplication complicates the accurate determination of the breakpoint. Regardless, our results align with prior research in this genomic locus, affirming the importance of investigating the genetic significance and phenotypic consequences of these identified duplication candidates (Boettger *et al*. 2012).

## 6 Conclusion

This study presents a comprehensive pipeline integrating deep learning techniques to expedite large-scale identification of CNVs within predefined genomic regions. Through application across five distinct use cases involving SVs within four genes implicated in NDD susceptibility, we showcase the adaptability and robustness of both our deletion and duplication models. By merging model predictions with domain-specific expertise, our approach significantly reduces the burden of manual validation, facilitating the identification of precise CNV candidates for subsequent downstream analyses or targeted validation using resource-intensive methods such as Polymerase Chain Reactions (PCR) or long-read sequencing.

## Supplementary Material

vbag205_Supplementary_Data

## Data Availability

Data used in the preparation of this article were obtained from GP2. Specifically we used Tier 2 data from GP2 (release 11: DOI 10.5281/zenodo.17753486). GP2 data can be accessed through AMP PD (https://amp-pd.org). This pipeline is implemented in Python and applied in Jupyter notebooks available for reference at: https://github.com/nvk23/CNV-Finder; DOI 10.5281/zenodo.19009012. Further documentation and example data is included in this repository with all pre-trained models saved in the CNV-Finder/ref_files/models folder.
