# Supplementary material for: CNV-Finder: streamlining copy number variation discovery
Source: Bioinform Adv. 2026 Jul 22;6(1):vbag205. doi: 10.1093/bioadv/vbag205 (PMC13503034; doi:10.1093/bioadv/vbag205)
Supplement: Supplementary file 1 [file vbag205_supplementary_data.zip › supplementary_tables.pdf]

Supplementary Tables

A. Positive Class – PRKN Deletions

Cohort	Diagnosis	Median Age	Males	Females	Total
APGS	PD	–	7	7	14
STELLENBOS	PD	55	8	3	11
BCM	PD	–	7	2	9
PAGE	PD	76	6	3	9
COPN	PD	67	6	0	6
PDGNRTN	PD	55	2	3	5
MDGAP-QSBB	PD	72	4	0	4
NZP3	PD	45	2	2	4
IPDGCAF-NG	PD	–	3	0	3
UMD	PD	–	0	2	2
BBDP	PD	77	1	0	1
KOC	PD	52	0	1	1
UMKLM	PD	54	1	0	1
					**70**

B. Positive Class – PRKN Duplications

Cohort	Diagnosis	Median Age	Males	Females	Total
PDGNRTN	PD	67	2	5	7
PAGE	PD	72	4	2	6
APGS	PD	–	3	2	5
COPN	PD	66	2	2	4
STELLENBOS	PD	60	1	2	3
UMD	PD	–	2	1	3
IPDGCAF-NG	PD	–	1	0	1
					**29**

C. Negative Class – No CNVs in PRKN

Cohort	Diagnosis	Median Age	Males	Females	Total
PPMI	PD	62	34	23	57
	Control	64	20	8	28
					**85**

Supplementary Table 1. Summary of training set from expert annotations of *PRKN* interval. (A) *PRKN* deletions (B) *PRKN* duplications (C) Negative samples with no CNVs.

Cohort	Diagnosis	Median Age	Males	Females	Unknown	Total
BBDP	Mix	76	153	95		248
	Control	85	120	106		226
	PD	78	111	43		154
	Undetermined-MCI	90	38	33		71
	DLB	80	14	5		19
	AD	72	0	1		1
CAT-PD	PD	62	149	190		339
	Control	53	172	150		322
	Other	39	2	1		3
	PSP	74	1	0		1
CORIELL	PD	63	2,784	1,657	2	4,443
	Control	67	1,779	2,249		4,028
	PSP	65	195	178		373
	MSA	61	27	26		53
	DLB	69	24	11		35
	Other	55	10	13		23
LCC	Control	52	86	129		215
	PD	67	79	69		148
MDGAP-KINGS	PD	78	65	34	4	103
	DLB	82	24	11		35
SYDBB	PD	63	60	28		88
	DLB	72	23	7		30
						**10,958**

Supplementary Table 2. Summary statistics of GP2 cohorts used in training our updated and final models. “Undetermined-MCI” represents undiagnosed mild cognitive impairment and “Prodromal” represents prodromal PD with no motor symptoms. “MSA” refers to Multiple System Atrophy.

A. PRKN Deletions

Cohort	Diagnosis	Median Age	Males	Females	Total
CORIELL	PD	55	99	51	150

B. PRKN Duplications

Cohort	Diagnosis	Median Age	Males	Females	Total
CORIELL	PD	59	28	10	38

C. Negative Class – No CNVs in PRKN

Cohort	Diagnosis	Median Age	Males	Females	Total
CORIELL	PD	64	2,621	1,523	4,144

Supplementary Table 3. Summary statistics of held-out expert annotations for validation of preliminary and updated models on CORIELL samples. CORIELL samples were incorporated in the training set of our final models.

Cohort	MLPA	Diagnosis	Median Age	Males	Females	Unknown	Total
**PFP**	With	PD	62	422	322		744
		Control	64	5	10		15
		Other	59	2	0		2
	Without	PD	65	327	208		535
		Control	60	33	52		85
		Other	63	35	34	2	71
		MSA	49	1	0		1
		PSP	73	1	0		1
						Subtotal	*1,454*
**STELLENBOS**	With	PD	67	274	196		470
		Control	24	2	1		3
	Without	PD	67	124	97		221
		Control	41	386	437		823
						Subtotal	*1,517*
						**Total**	**2,971**

Supplementary Table 4. Summary statistics of PFP and STELLENBOS samples with MLPA data for prediction validation.

Data Type	Diagnosis	Median Age	Males	Females	Total
Short-read WGS	PD	65	154	73	227
	Control	64	71	37	108
	Other	66	2	0	2
				**Total**	**337**
Long-read WGS	PD	68	11	6	17
	Control	66	2	5	7
	Prodromal	67	0	1	1
	Other	52	1	0	1
				**Total**	**26**

Supplementary Table 5. Summary of PPMI extension data available for prediction validation. No samples described here overlap with any PPMI samples incorporated in the models' training sets.

Cohort	Diagnosis	Median Age	Males	Females	Unknown	Total
MDGAP-DEMENTIA	AD	59	52	55	3	110
	PSP	71	62	32		94
	FTD	58	42	25	1	68
	Other	67	21	14	7	42
	DLB	61	28	13		41
	MSA	65	8	7		15
	CBD/CBS	67	4	4		8
	PD	51	0	1		1
REGARDS	AD	75	60	40		100
	Undetermined-Dementia	75	14	13		27
						**506**

Supplementary Table 6. Summary statistics of additional NDD phenotype cases.

Feature Name Calculated per Window	Description
Dosage Full	Ratio of CNV candidates in window to total number of CNV candidates across full region of interest
CNV-specific Dosage Full	Ratio of specific CNV candidates (either deletions or duplications) in window to total number of CNV candidates across full region of interest
CNV-specific Dosage Interval	Ratio of specific CNV candidates (either deletion or duplication) in window to total number of all variants in that window
BAF Std. Dev.	Standard deviation of B Allele Frequencies within window
Mid-BAF Std. Dev.	Standard deviation of B Allele Frequencies (≥0.15 & ≤0.85) within window
LRR Std. Dev.	Standard deviation of Log R Ratio within window
BAF IQR	Interquartile range of B Allele Frequencies within window
Mid-BAF IQR	Interquartile range of B Allele Frequencies (≥0.15 & ≤0.85) within window
LRR IQR	Interquartile range of Log R Ratio within window
Avg. BAF	Average B Allele Frequencies within window
Avg. Mid-BAF	Average B Allele Frequencies (≥0.15 & ≤0.85) within window
Avg. LRR	Average Log R Ratio within window
**Feature Combination**	**Features Included**
Combination 1	Dosage Interval, CNV-specific Dosage Full, Avg. BAF, Avg. LRR
Combination 2	Dosage Interval, Dosage Full, CNV-specific Dosage, BAF SD, LRR SD, BAF IQR, LRR IQR, Avg. BAF, Avg. LRR
Combination 3	Dosage Interval, Dosage Full, CNV-specific Dosage Full, CNV-specific Dosage Interval, BAF SD, LRR SD, BAF IQR, LRR IQR, Avg. BAF, Avg. Mid BAF, Avg. LRR
Combination 4	Dosage Interval, Dosage Full, CNV-specific Dosage Full, CNV-specific Dosage Interval, BAF SD, Mid BAF SD, LRR SD, BAF IQR, Mid BAF IQR, LRR IQR, Avg. BAF, Avg. Mid BAF, Avg. LRR
Combination 5	Dosage Interval, Dosage Full, CNV-specific Dosage Full, BAF SD, Mid BAF SD, LRR SD, BAF IQR, Mid BAF IQR, LRR IQR, Avg. BAF, Avg. Mid BAF, Avg. LRR
Combination 6	Dosage Interval, CNV-specific Dosage Full, BAF SD, Mid BAF SD, LRR SD, BAF IQR, Mid BAF IQR, LRR IQR, Avg. BAF, Avg. Mid BAF, Avg. LRR

Supplementary Table 7. Descriptions of features and tested combinations used in model training. “Avg.” stands for average, “IQR” represents interquartile range, and “SD” refers to standard deviation.

Statistical Metric	Description
Area Under the Curve (AUC)	Demonstrates performance of a classification model by representing how well the model can separate and distinguish the classes. In our case, we are evaluating how accurately our model outputs a predicted value above 0.5 when a CNV exists in the region of interest for each sample.
Brier Score	Evaluates the accuracy of predicted probabilities of an event with a range of 0 to 1, with a score of 0 representing perfect accuracy. This score reveals the accuracy of our models' predicted values of a CNV existing when compared to the actual existence of the SV. It allows us to see if the predicted value output by the model correlates with the strength of CNV candidates, meaning that the most easily observable deletions and duplications should receive predicted probabilities above 0.9.
Positive Predictive Value (PPV)	Represents the likelihood that a sample with a predicted value of 0.5 from our models would have a CNV present in the region of interest.
Jaccard Similarity Score	Measures the similarity between two datasets by dividing the intersection of both sets by their union. A score of 1.0 implies that two datasets include the same members; therefore in our "Duplications within the 17q21.31 Region" analysis, the score of 0.94 implies that the H2-carrier status was "Yes" in almost every instance that the presence of an obvious duplication in the 17q21.31 region was "Yes."

Supplementary Table 8. Descriptions of statistical metrics used in assessing model performance.

		Deletions		Duplications	
**Cohort**	**CNV Size Range**	**PennCNV**	**CNV-Finder**	**PennCNV**	**CNV-Finder**
STELLENBOS	< 10 kb	2 (6%)	2 (7%)	2 (29%)	0 (0%)
	10 – 50 kb	14 (39%)	10 (33%)	0 (0%)	0 (0%)
	50 – 100 kb	11 (31%)	9 (30%)	0 (0%)	0 (0%)
	100 – 500 kb	8 (22%)	8 (27%)	4 (57%)	4 (80%)
	> 500 kb	1 (3%)	1 (3%)	1 (14%)	1 (20%)
PFP	< 10 kb	1 (2%)	0 (0%)	0 (0%)	0 (0%)
	10 – 50 kb	11 (24%)	5 (14%)	0 (0%)	0 (0%)
	50 – 100 kb	5 (11%)	4 (11%)	0 (0%)	0 (0%)
	100 – 500 kb	27 (60%)	27 (73%)	14 (100%)	12 (100%)
	> 500 kb	1 (2%)	1 (3%)	0 (0%)	0 (0%)

Supplementary Table 9. Size distribution of confirmed *PRKN* CNV nominations detected by PennCNV and CNV-Finder across PFP and STELLENBOS cohorts. Values represent the number of confirmed CNVs within each size range, with the percentage of total confirmed calls for that CNV type in the respective cohort shown in parentheses. Detection was concordant at 50 kb and above, while differences were confined to the smaller bins (<10 kb, and 10–50 kb deletions in PFP), reflecting the resolution limits of array-based detection at low probe density.
